# Inflammation in Cancer: Part of the Problem or Part of the Solution?

**DOI:** 10.1155/2019/5403910

**Published:** 2019-02-07

**Authors:** Monica Neagu, Donato Zipeto, Iulia D. Popescu

**Affiliations:** ^1^“Victor Babes” National Institute of Pathology, Bucharest, Romania; ^2^University of Verona, Verona, Italy; ^3^University of Pittsburgh, Pittsburgh, USA

With the advent of the new era of immune therapy, new aspects of inflammation as a process involved in therapy, in tumorigenesis, and in several other human pathologies have been highlighted [1]. This special issue attempted to cover as many of these inflammation aspects in original papers and in extended reviews.

It is possible to trait two distinct sections discussing inflammation aspects; a series of papers focusing on oncology and a series of papers focusing on other inflammation-related pathologies. From the first series of papers, M. Tampa et al. review the inflammatory markers of oral squamous cell carcinoma highlighting the main markers of inflammation that could improve early diagnosis. In esophageal squamous cell carcinoma cells, M. Wang et al. show that in *in vitro* studies, PAR4 can have a tumor suppressor role and can be used as a future therapeutic target. In endometrial cancer, S. Gu et al. show that tumor-associated macrophages are predominantly type 2 (M2) that contribute to the progression of this type of cancer and that the anti-CD27 therapy could have an antitumoral effect. Similarly, tumor-associated macrophages are reviewed by H. Degroote et al. in another type of cancer, hepatocellular carcinoma. The preclinical evolution and hitherto clinical trials for TAM-targeted therapy in HCC have been highlighted.

A syndrome associated with cancer progression is cachexia, reviewed by E. Manole et al. The paper is describing several myokines produced and released by myocytes that can become potential biomarkers and future therapeutic targets.

As already mentioned, immune therapy would be probably one of the major breakthroughs in oncology. C. Vajaitu et al. review checkpoint inhibitor new treatments and the role of inflammation in these therapies presenting biomarkers that could predict efficacy and immune therapy resistance.

A. Calinescu et al. reviewed an inflammatory-related protein, carcinoembryonic antigen-related cell adhesion molecule 1, and its involvement in malignancies. The paper shows its importance as a prognostic factor in oncology and a future target specific cancer therapy.

The second section of the special issue consists of reviews and original papers evaluating inflammation in various nononcological diseases. A. Pedrinolla et al. evaluate proinflammatory markers of age-related obesity. M. Bucur et al. highlight in their original paper that there are clear profibrotic and antifibrotic factors expression differences in oral mucosa and skin scars.

M. Dobre et al. show that differences found in various transcript levels of inflammatory molecules could aid the differential diagnosis between ulcerative colitis and Crohn's disease. Chronic kidney disease could benefit from current proteomic approaches, as S. Mihai et al. are describing the inflammasomes and gut microbiota dysbiosis involvement in this disease and moreover in the renal malignancy.

An in vivo animal model is shown by E. Codrici et al. where a caveolin-1-knockout mouse is thoroughly characterized and presented as a good inflammatory disease model. Another original paper by V.M. Anghelescu et al. shows the inflammatory pattern evaluation in animal models associated with various bone implants. Last, but not least, S.R. Georgescu et al. revise the traits of chronic inflammation in HPV infection that can lead to tumorigenesis.

We are very satisfied that our subject resulted in so many valuable papers, and we would like to thank all the authors who submitted their work for consideration to this special issue. Without their effort, this special issue would have not taken place. Editors would like to thank the reviewers who thoroughly revised the papers and provided important suggestions that significantly improved the papers.
